# Phytochemical Characterization, Antioxidant, and Antimicrobial Activity of the Vegetative Buds from Romanian Spruce, *Picea abies* (L.) H. Karst.

**DOI:** 10.3390/molecules29092128

**Published:** 2024-05-03

**Authors:** Roxana Colette Sandulovici, Mona Luciana Gălăţanu, Luiza Mădălina Cima, Emilia Panus, Elena Truţă, Carmen Marinela Mihăilescu, Iulian Sârbu, Daniel Cord, Mirela Claudia Rîmbu, Ştefan Alexandru Anghelache, Mariana Panţuroiu

**Affiliations:** 1 Faculty of Pharmacy, Titu Maiorescu University, 16 Sincai, Boulevard, 040314 Bucharest, Romania or roxana.sandulovici@prof.utm.ro (R.C.S.); luiza.cima@prof.utm.ro (L.M.C.); helen_truta@yahoo.com (E.T.); carmen_mihail28@yahoo.com (C.M.M.); iulian.sarbu@prof.utm.ro (I.S.); cord_daniel@hotmail.com (D.C.); mirela.rimbu@prof.utm.ro (M.C.R.); stefan.anghelache@s.utm.ro (Ş.A.A.); rdpharma1@yahoo.com (M.P.); 2 Microbiology and Molecular Biology Laboratory, Public Health Constanta, 900587 Constanta, Romania; temilia2@yahoo.com; 3 National Institute for Research and Development in Microtechnologies, 126A. Erou Iancu Nicolae Street, 72996 Bucharest, Romania; 4 National Agency for Medicines and Medical Devices of Romania, Stefan Sanatescu Street 48, 011478 Bucharest, Romania

**Keywords:** *Picea abies* vegetative buds, HPLC, essential oil, unpolluted area, antioxidant capacity, antimicrobial activity

## Abstract

This study aims to investigate the vegetative buds from *Picea abies* (spruce), naturally found in a central region of Romania, through a comprehensive analysis of the chemical composition to identify bioactive compounds responsible for pharmacological properties. Using HPLC/derivatization technique of GC-MS and quantitative spectrophotometric assays, the phenolic profile, and main components of an ethanolic extract from the buds were investigated. The essential oil was characterized by GC-MS. Moreover, the antioxidant activity with the DPPH method, and the antimicrobial activity were tested. Heavy metal detection was performed by graphite furnace atomic absorption spectrometry. The main components of the alcoholic extract were astragalin, quercetin, kaempferol, shikimic acid, and quinic acid. A total content of 25.32 ± 2.65 mg gallic acid equivalent per gram of dry plant (mg GAE/g DW) and of 10.54 ± 0.083 mg rutin equivalents/g of dry plant (mg RE/g DW) were found. The essential oil had D-limonene, α-cadinol, δ-cadinene, 13-epimanool, and δ-3-carene as predominant components. The spruce vegetative buds exhibited significant antioxidant activity (IC50 of 53 μg/mL) and antimicrobial effects against *Staphylococcus aureus*. Furthermore, concentrations of heavy metals Pb and Cd were below detection limits, suggesting that the material was free from potentially harmful contaminants. The results confirmed the potential of this indigenous species to be used as a source of compounds with pharmacological utilities.

## 1. Introduction

The Romanian flora is an important source of medicinal plants with traditionally uses in the folk medicine.

*Picea abies* (L.) H. Karst., known as spruce, or Norway spruce, is a coniferous tree belonging to *Pinaceae* family, wild growing in the North hemisphere. About 23% of Romanian forests are of spruce, usually being found at 600–1700 m altitude [1,2]. This species has a high economic value, as raw material for making cellulose, paper, and wood products. In recent years, spruce is also used in alimentary products, such as jams, syrups, and beverages [3,4].

The spruce tree has a pyramidal shape, and a high stem, up to 50 m length. The waxy dark-green, acicular leaves have 4 edges, a rhomboidal section, and a sharp, pointed tip. The female cones are rhomboid, brown, elongated, and pedantic. The seeds are winged and attached on the upper face of the female cone’s scales [1,5].

Different parts of spruce such as needles, vegetative buds, and bark are used in traditional medicine. They contain important biocompounds, as carbohydrates, terpenoids, resins, fatty acids, phenolic compounds (polyphenolic acid, flavonoids, stilbens, lignans, and tannins), and piperidinic alkaloids [1,6,7]. Spruce is also a source of Vitamin C, E, and minerals (calcium, potassium, zinc) [8]. Some of these substances serve as source of energy for the tree, some have an important role in physiological functions (metabolism, growing, dormancy or temperature regulation), while others are used for defending against the oxidative stress, and pathogens.

Few studies on the essential oil distilled from spruce buds and needles shown that is has monoterpenoids (α-pinene, camphene, limonene, and myrcene), oxygenated monoterpenes (bornyl acetate), sesquiterpenoids (β-caryophyllene germacrene D, δ-cadinene, and muurolene), oxygenated sesquiterpenes (caryophyllene oxide, germacrene-4-ol, nootkatone, nerolidol, farnesol, cadinol, and muurolol), diterpenoids (abietadiene and manool) as main components [9,10]. However, it is known that the content of the essential oils can vary depending on genotype, geographic origin, pollution, the type of plant material, methods of obtaining and analyzing. 

Spruce is traditionally used for its anti-inflammatory and antiseptic action, in respiratory and skin diseases as different pharmaceutical preparations: syrup, tea, creams, ointments, and baths [4]. Recent clinical studies have shown antibacterial and antifungal properties [11,12], wound healing [13], immunological, anti-inflammatory and antioxidant effects [14,15,16] and even antitumoral action [17,18,19] for the spruce, due to its bioactive compounds. 

Spruce, a coniferous species widespread in mountainous areas, is frequently used in environmental research because of its exceptional ability to accumulate pollutants over long periods of time. This species is particularly suitable for monitoring studies due to its wide distribution, adaptation to different habitats and annual needle growth, which allows the assessment of chemical element concentrations over different age ranges. In addition, Norway spruce has a remarkable ability to absorb various substances, including sulfur and heavy metals, from atmospheric emissions [20,21,22,23].

Certain trace elements, including copper, manganese, iron, and zinc, are essential for plants and play an important role in metabolic processes [24,25]. However, when these elements are present at high levels in the soil, they become toxic to both plants and micro-organisms. On the other hand, lead and cadmium, which are heavy metals that are not necessary for the functioning of plant organisms [26,27], can also be harmful. Therefore, in order to ensure the quality and safety of future plant-based pharmaceutical preparations, the presence of certain heavy metals (Cd, Pb) has been studied. 

The aim of our research was to identify and analyze the most important bio compounds from the vegetative buds of the Romanian spruce, and to determine some of pharmacological actions such as antioxidant and antibacterial capacity. 

## 2. Results

### 2.1. Total Phenolic and Flavonoid Content

Spectrophotometric methodology was used to evaluate the content of phenolics and flavonoids in spruce buds (*Picea abies*) from Romania. Colorimetric tests were useful for the initial screening of the content of phenolic compounds in the extract, but for a more detailed and accurate evaluation, we used more precise and specific techniques, such as liquid chromatography with detection by mass spectrometry (HPLC-MS) or other advanced analytical methods [28,29,30]. The results obtained by applying the spectrophotometric methodology allowed the qualitative and quantitative estimation of the main groups of biologically active compounds from the buds of the *Picea abies* species that grows in Romania. The total content of polyphenols was 25.32 ± 2.65 mg GAE/g DW (gallic acid equivalent) and 24.39 ± 1.76 mg CAE/g DW (caffeic acid equivalent). The flavonoid content was found to be 10.54 ± 0.083 mg RE/g DW (rutin equivalent). The amounts of phenolic compounds detected in the samples are shown in Table 1. These results indicate the presence of a significant content of polyphenols in spruce buds. The level of flavonoids is also significant, indicating the presence of important bioactive compounds [30,31,32].

### 2.2. HPLC Analysis

**Figure 1:**
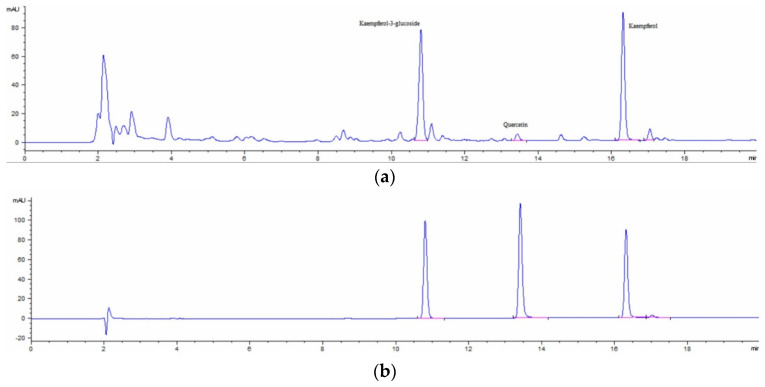
HPLC chromatograms of the hydroalcoholic extract spruce buds (**a**) and the reference solution (**b**).

The chromatograms of the flavonoid compounds from the *P. abies* bud extract and the reference solution are presented in Figure 1a,b.

HPLC analysis provided more detailed information on specific compounds in spruce buds. Phenolic compounds were identified based on retention times and UV spectra compared to known standards and literature references. Astragalin, quercetin, kaempferol, and flavonoids, known for their antioxidant and health-promoting properties, were identified. The HPLC method determined a content of 11.30 mg astragalin/g and 10.00 mg kaempferol/g plant product, at the following retention times: astragalin—10.8 min; kaempferol16.31 min. The results suggest that spruce buds are a rich source of phenolic compounds and flavonoids, which have the potential to provide significant health benefits.

### 2.3. GC-MS Analysis

**Figure 2:**
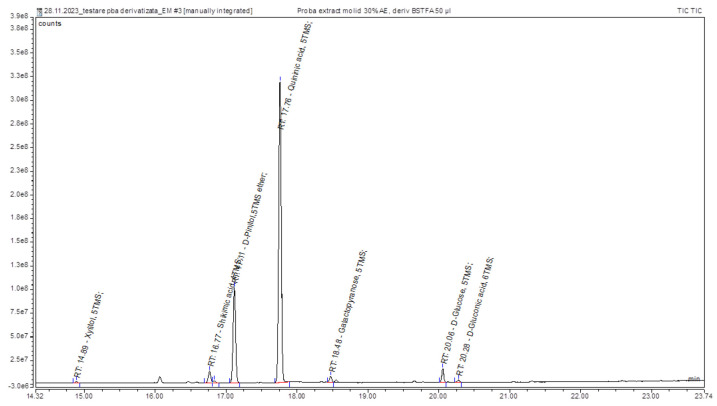
TIC Chromatogram resulting from the analysis of a derivatized sample of spruce (*Picea abies*) bud extracts: (1) xylitol, (2) shikimic acid, (3) pinitol, (4) quinic acid, (5) galactopyranose, (6) D-glucose, (7) D-glucose, and (8) gluconic acid.

The derivatization technique combined with GC-MS was employed to identify compounds in the spruce extract, as shown in Figure 2. The retention times of the silylated phenolic compounds in the examined plant extract are listed in Table 2. The derivatization process involves reacting phenolic compounds with a silylating reagent, such as trimethylsilyl (TMS) derivatives, to form silylated derivatives. These derivatives exhibit improved chromatographic properties, enhancing separation and detection in GC-MS analysis. The data obtained allowed us to observe the presence of the following compounds in the spruce bud extract: shikimic acid, quinic acid, xylitol, D-pinitol, galactopyranose, glucose, and gluconic acid. The different compounds identified, including organic acids, polyols, and carbohydrates, suggest that these extracts may have a wide range of applications [33].

### 2.4. Heavy Metal Detection

The performance parameters of heavy metal detection are shown in Table 3. Romanian vegetative buds of spruce showed no detectable amounts of the heavy metals Cd and Pb (undetectable), which indicated that they were harvested from an unpolluted area.

### 2.5. Essential Oil Analysis

By hydro distillation in a closed-circuit Neo-Clevenger apparatus of the spruce vegetative buds, an essential oil of pale, slightly yellow color and a fresh, herbal, woody-lemon scent was obtained. The essential oil yield was of 0.455 ± 0.12% (mL/100 g dried plant).

The essential oil composition is shown in Table 4 and Figure S1 in Supplementary Material. A total of 20 compounds were identified, accounting for 98.74% of the essential oil composition.

The essential oil from the vegetative buds of Romanian spruce contained monoterpenoids (D-limonene, δ-cadinene, δ-3-carene, β-pinene, myrcene, camphene), sequiterpenoids (caryophyllene, δ-cadinene, longifolene), sesquiterpenic alcohols (α-cadinol, τ-muurolol, τ-cadinol), diterpenic alcohols (13-epimanool), and diterpenic aldehydes (ent-kaurenal). The dominant components were D-limonene (40.43%), α-cadinol (12.53%), δ-cadinene (9.95%),13-epimanool (8.52%), and δ-3-carene (4.32%). Among these constituents, ent-kaurenal is discovered for the first time in the spruce essential oil.

### 2.6. Antioxidant Activity

In this study, the antioxidant activity of the spruce alcohol extract was assessed using various volumes of the extract, each containing 253.233 μg/mL of total polyphenols (TPC) expressed in gallic acid equivalents, via the 2,2-diphenyl-1-picrylhydrazyl radical (DPPH) scavenging assay. The IC50 value, representing the concentration of the extract necessary to neutralize 50% of DPPH radicals, was determined as a measure of antioxidant potency. The obtained IC50 value was 53 μg/mL.

This result indicates that at a concentration of 53 μg/mL, the spruce alcohol extract was able to scavenge 50% of the DPPH radicals present in the assay solution. A lower IC50 value suggests a higher antioxidant activity, indicating that the extract exhibited potent antioxidant properties. Overall, these findings demonstrate the effectiveness of the spruce alcohol extract in combating oxidative stress by neutralizing free radicals, which may have implications.

The results revealed a direct correlation between the antioxidant actions observed and the concentration of secondary metabolites in the sample.

### 2.7. Antibacterial Activity

**Figure 3:**
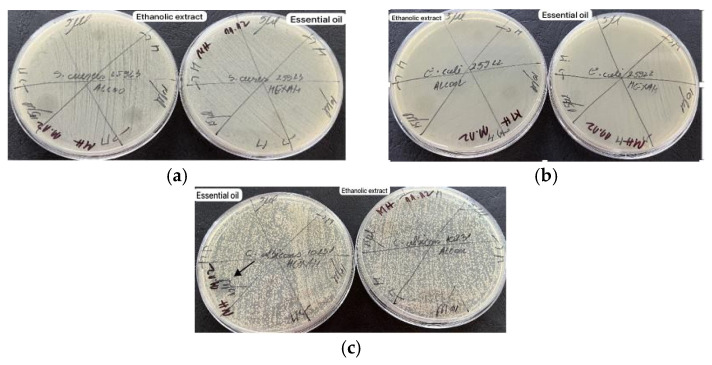
Antimicrobial activity for reference strains with Kirby-Bauer diffusimetric method: (**a**) *S. aureus* ATCC 25923, (**b**) *E. coli* ATCC 25922; (**c**) *C. albicans* ATCC 10231.

According to Figure 3, the diffusimetric method showed that only the volatile oil exhibited microbial activity for *Candida albicans* among the two samples tested, especially at a volume of 15 µL.

**Figure 4:**
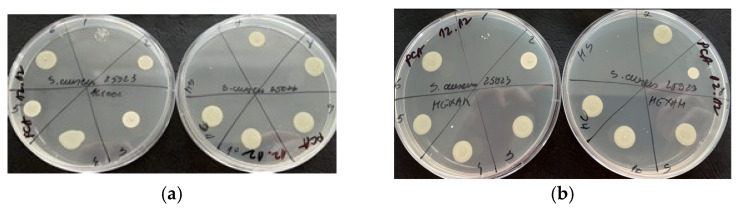
Antimicrobial activity by the microdilution method for reference strains *S. aureus* ATCC 25923 (**a**)—alcoholic extract and (**b**)—essential oil.

**Figure 5:**
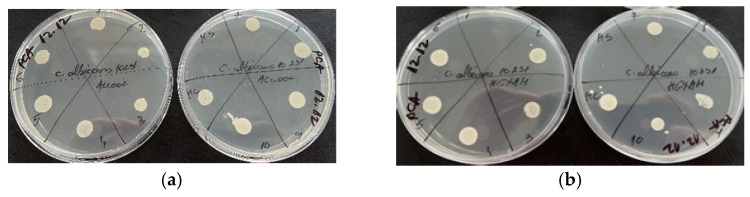
Antimicrobial activity by the microdilution method for reference strains *C. albicans* ATCC 10231 (**a**)—alcoholic extract and (**b**)—essential oil.

**Figure 6:**
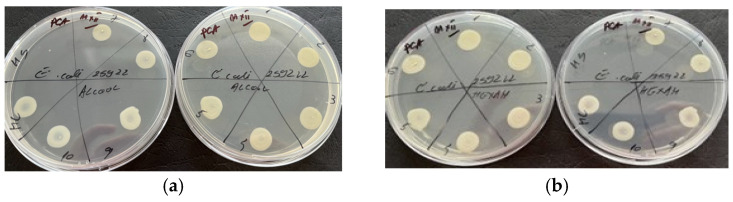
Antimicrobial activity by the microdilution method for reference strains *E. coli* ATCC 25922 (**a**)—alcoholic extract and (**b**)—essential oil.

Based on the results obtained by the microdilution method, it was observed that both samples tested exhibited antimicrobial properties against *S. aureus*, a Gram-positive bacterium (Figure 4). This suggests that both samples contain compounds that are effective against this type of bacteria. Additionally, it was observed that only the volatile oil showed antimicrobial activity against *C. albicans*, as shown in Figure 5. It should be noted that antimicrobial activity was observed at a volume of 20 µL for both *S. aureus* and *C. albicans*. None of the plant materials showed antimicrobial activity on Gram negative bacteria (Figure 6). This could imply that the antimicrobial compounds present in the samples are more effective against Gram-positive bacteria and fungi and may not have activity against Gram-negative bacteria.

**Figure 7:**
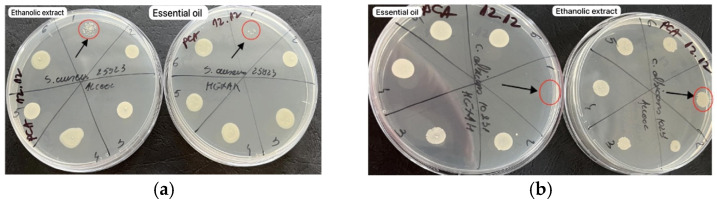
Antimicrobial activity of alcohol extract versus essential oil of *Picea abies* buds for reference strains: (**a**) *S. aureus* ATCC 25923; (**b**) *C. albicans* ATCC 10231.

Both samples exhibited antimicrobial activity, indicating the presence of compounds capable of inhibiting the growth of this Gram-positive bacterium. However, the activity against *Candida albicans* was observed only in the volatile oil sample (Figure 7).

## 3. Discussion

In this research we determined the main phytochemicals from a specific plant material, as vegetative buds, collected from the Romanian species of spruce. We have analyzed the antioxidant activity, their antimicrobial action, and the grade of pollution of the plant from the native area.

The significant levels of polyphenols and flavonoids detected in the spruce buds indicate the presence of important bioactive compounds. Polyphenols and flavonoids are well-known for their antioxidant properties and potential health benefits. These compounds have been associated with various biological activities, including anti-inflammatory, antimicrobial, and anticancer effects. The HPLC analysis of flavonoid compounds in *Picea abies* (spruce) bud extract revealed the presence of several important phenolic compounds, including astragalin, quercetin and kaempferol. The presence of different compounds in the spruce bud extract underlines its chemical complexity and potential versatility. Among these compounds, shikimic acid stands out as a particularly important component. Shikimic acid, also known as 3,4,5-trihydroxycyclohex-1-ene-1-carboxylic acid, plays a crucial role in plant biosynthesis and serves as a precursor for the synthesis of numerous chemicals [34]. One of the most notable applications of shikimic acid is its role as a precursor in the synthesis of neuraminidase inhibitors, which are drugs used in the treatment and prevention of influenza A and B viruses. This application has gained widespread recognition during influenza outbreaks, in especially due to the production of the antiviral drug oseltamivir, which is synthesized using shikimic acid [35,36]. Furthermore, shikimic acid derivatives exhibit various pharmacological effects, including anti-inflammatory, antibacterial and antioxidant properties. These properties contribute to the compound’s importance in medical applications beyond antiviral treatments. For example, its anti-inflammatory properties make it potentially valuable in alleviating inflammatory conditions, while its antibacterial effects suggest utility in fighting bacterial infections [37,38,39]. Overall, the presence of shikimic acid in spruce bud extract highlights its potential for various applications, not only in pharmaceuticals, but also in fields such as supplements and cosmetics.

It appears that the concentration of shikimic acid in spruce needles fluctuates throughout the year, with the highest concentrations in needles harvested in November and April (94.7 mg/g and 88.69 mg/g, respectively), according to Ozhimkova et al. This suggests that the November-April period is optimal for spruce needle harvesting in order to obtain the highest amount of shikimic acid produce shikimic acid [40].

Cadmium (Cd) and lead (Pb) were not detected in the Romanian spruce buds. These heavy metals are often released into the environment via vehicles, and over time dust particles or precipitation can deposit contaminants, causing soil and plant pollution. Interaction between trace heavy metal pollutants and plants starts in the roots, especially in the root endoderm, where contaminants are either filtered out (Pb) or absorbed (Cd). These pollutants are then transported to aboveground organs, eventually accumulating in foliage and cones [41,42]. Mobile metal contaminants enter leaf tissues through the vein system and accumulate along apoplastic and symplastic water and nutrient pathways, eventually reaching water evaporation sites [43]. However, if necessary, these contaminants can be stored in tissue-safe compartments away from vulnerable areas of uptake and transport. Accumulation of heavy metals, especially Cd, induces oxidative stress, triggers degenerative processes and results in necrosis in the foliage, especially in the lower leaf section, independent of photo-oxidative stress [44,45].

Today, because of strict pollution regulations, the levels of cadmium and lead discharged into the environment are steadily decreasing. As a result, spruce buds collected from an ecologically clean location, away from industrial areas and heavily trafficked roads, showed metal concentrations below detection limits, which make them a good source of medicinal plant material.

An important aspect of the therapeutic potential of this species is the presence of the essential oil. The amount of essential oil found in the vegetative buds of spruce (0.455 ± 0.12%) correspond with that of spruce essential oil from Lithuania (0.41–0.52%), Latvia (0.36–0.55%), and Slovenia (0.02–0.34%). The percentage is However, lower than the essential oil of spruce from other geographical regions in Romania (1.01–1.02%), but this can be explained by the different type of vegetal material they have used, such as mature needles, or bark. [8,10,46,47,48].

D-limonene was the major compound in the essential oil (40.43%), and its concentration was higher than reported by other authors in spruce (21.14%, respectively 12.98%) [8,10]. D-limonene is a monoterpenoid with many pharmacological effects, such as chemo preventive and anticancer activity (breast and colorectal) [19], dissolving cholesterol gallstones [49], ameliorating the gastroesophageal reflux [50], and it is appreciated by the Code of Federal Regulations as generally recognized as safe (GRAS). D-limonene has also an important role in plant resistance to pathogenic microorganisms. Silva et al. (2020) showed that D-limonene, as the main component from the essential oil of *Eucalyptus* leaves is responsible for the inhibition of pathogenic fungi that can affect this species [45]. The mechanism of defense involves disruption of the unsaturated fatty acids from the pathogen’s membranes [51,52,53].

Another dominant component of the essential oil is α-cadinol (12.53%), which possesses hepatoprotective and antifungal action [54,55]. δ-cadinene is the third major compound in this essential oil (9.95%), a sesquiterpenoid with antiproliferative, apoptotic and anticancer action proved on human ovary cancer cell lines [56]. Other interesting and important constituents from the essential oil are 13-epimanool, a diterpenic alcohol with antifungal and anticancer activities, demonstrated on human leukemic cells and human neuroblast cells [57];caryophyllene, a bicyclic sesquiterpenoid, an agonist of cannabinoid receptors CB2, used in ameliorating chronic inflammatory pains [58] and with anticancer properties; δ-3-Carene, wich has sleep-enhancing and anxiolytic actions [59]; τ-cadinol, that has a muscle relaxing effect [60]; β-pinene, a monoterpenoid widely found in conifers with anxiolytic, gastroprotective, antibiotic actions [61], and ent-kaurenal, precursor of the plant hormone gibberellin A_12_, with antibacterial, antitumor and anti-inflammatory effects [62].

The composition of the essential oil from the Romanian spruce is in accordance with the data from literature, and the variations are due to plant material, geographical regions, genotype, or extraction method. The vegetative buds are young parts of a plant, with a reduced capacity to produce secondary metabolites, used for defense and metabolic activities, such as those founds in the mature tissues. The bio compounds found in the essential oil of the spruce vegetative buds gives a complex phytochemical profile, with protective, anticancer, antimicrobial, and anti-inflammatory potential.

Screening for antioxidant potential is important for assessing the bioactive value of plant extracts. Spruce bud extract has demonstrated the ability to reduce the stable free radical DPPH (2,2-diphenyl-1-picrylhydrazyl) to yellow diphenylpicrylhydrazine. This implies that the extract contains active constituents capable of donating hydrogen to free radicals, proving antioxidant properties, attributed to the presence of flavonoids and phenolic compounds. Polyphenols, which include flavonoids among other compounds, are recognized for their powerful antioxidant properties, attributed to their ability to scavenge free radicals, and counteract oxidative stress in the body. These results contribute to the understanding of the antioxidant properties of spruce extracts and their potential applications in various industries, including pharmaceuticals, cosmetics, and food. A single method of analysis, however, may not provide a comprehensive understanding of the antioxidant activity of the extract we studied. For a more precise approach, it is necessary to use other assays in the future to capture the full spectrum of antioxidant activity [10,11,14,63].

Numerous authors have conducted studies on the antimicrobial properties of essential oil derived from various members of the *Pinaceae* family [6,64,65]. The vegetative buds of Romanian spruce showed a high antibacterial activity on Gram-positive bacteria. The results obtained from the microdilution method reveal significant antimicrobial properties in the samples tested against *Staphylococcus aureus.* The absence of antimicrobial activity against *Escherichia coli*, a Gram-negative bacterium, in all tested samples could be attributed to the structural differences in the cell wall between Gram-positive and Gram-negative bacteria. Gram-negative bacteria possess an outer membrane that serves as an effective barrier against the internalization of active compounds present in the extracts. This outer membrane, composed of lipopolysaccharides, contributes to a more negatively charged and hydrophilic surface compared to the cell wall of Gram-positive bacteria, which primarily consists of peptidoglycan. This structural disparity may limit the permeability of lipophilic substances, thereby reducing the efficacy of the extracts against Gram-negative bacteria like *E. coli* [66]. It can be noticed that volatile oil potentiated antimicrobial activity for both *S. aureus* and *C. albicans.* The observed potentiation of antimicrobial activity by the volatile oil suggests that certain components within the volatile oil enhance the antimicrobial effects of the extracts. This enhancement could be due to synergistic interactions between different compounds present in the volatile oil, leading to increased efficacy against the tested microorganisms. Overall, the findings highlight the potential of the tested extracts, particularly the volatile oil, as sources of antimicrobial agents that can be used in various pharmaceutical products. Further extended studies on more strains (bacteria, fungi, viruses) are needed to be realized to have a larger view of Romanian spruce antimicrobial activity.

Studies have shown that polyphenols and polyphenol-rich extracts have indeed demonstrated significant antimicrobial activity against a wide range of microorganisms, including bacteria, fungi, viruses, and parasites. This antimicrobial activity can be attributed to various mechanisms: disruption of cell membranes, inhibition of enzymes, antioxidant properties, modulation of host immune response [67].

Quinic acid (QA) and shikimic acid (SA), two kinds of natural organic acids, have been reported to exhibit potent antibacterial activity against *Staphylococcus aureus*. The study by Bai J et al. investigated the effects of quinic acid (QA) and shikimic acid (SA) on the cellular functions of *Staphylococcus aureus* and showed that both acids remarkably reduced the DNA content of *S. aureus* and interacted directly with its genomic DNA resulting in potent antibacterial activity The results of the research indicate that despite their similar chemical structures, quinic acid (QA) and shikimic acid (SA) have distinct effects on cellular functions. Furthermore, the findings suggest that both SA and QA possess antibacterial properties that could be harnessed for food preservation purposes [68].

Moreover, some studies discuss the potential health benefits and antibacterial activity of *Picea abies* (spruce) buds, focusing on the compound 1,6-dehydropinidine and its interactions with other bioactive compounds present [69,70]. *P. abies* buds, which contain high concentrations of 1,6-dehydropinidine, are traditionally considered to be beneficial to health, particularly in treating symptoms such as cough [71]. The study by Virjamo et al. regarding antibacterial activity found that despite the presence of 1,6-dehydropinidine it showed only mild antibiotic activity against the Gram-positive bacterium *Streptococcus equi*. This indicates that 1,6-dehydropinidine may not be the main compound responsible for the observed antibacterial effects [72]. Further research is needed to understand the interaction between 1,6-dehydropinidine and other bioactive compounds in.

Studies such as the one conducted by Fyhrquist et al. have indeed shed light on the promising antibacterial and antifungal effects of epidihydropinidine, a major piperidine alkaloid found in Norway spruce needles and bark. These findings underscore the complex interplay between toxicity and antimicrobial activities associated with piperidine alkaloids [70].

It is important to note that piperidine alkaloids have been reported to exhibit acute toxicity in adult livestock species and may have teratogenic effects [73]. Additionally, studies suggest the possibility of synergistic or additive interactions between alkaloids, phenolics, and terpenes, which could further enhance the antibacterial activity of *Picea abies* against microorganisms, including phytopathogenic bacteria.

There is a need for further studies on potential toxicity issues arising from the presence of piperidine alkaloids. This highlights the importance of conducting further research to understand in detail the safety profile of spruce-derived compounds and their potential applications in various contexts.

## 4. Materials and Methods

### 4.1. Plant Material and Extraction

**Figure 8:**
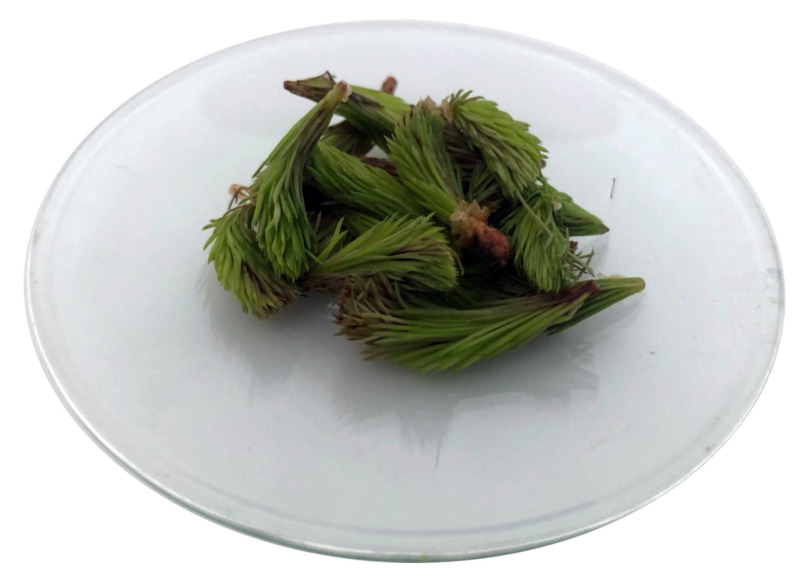
The fresh buds of *Picea abies* (L.) H. Karst., spruce (Source: M. Panţuroiu).

The vegetative buds of Romanian spruce (*Picea abies*) were collected from various trees located in Arges county, village Stoenesti, with geographical coordinates 45°16′33.4″ N and 25°13′20.6″ E, in April 2023. The buds were harvested from branches situated at 1.5–2.5 m on the central stem of the trees., without impurities such as fungi, insects, mud, dried parts (Figure 8). The plant material was harvested from an ecologically clean area, away from industrial sites and the edge of heavy traffic roads, ensuring minimal environmental contamination. A voucher specimen of the specie was deposited at “Dimitrie Brândză” Botanical Garden, Bucharest, Romania, with number 410415.

To determine the loss on drying, to obtain the essential oil by hydro distillation and to detect the heavy metals we have used the spruce buds in a fresh state, immediately after harvesting.

**Figure 9:**
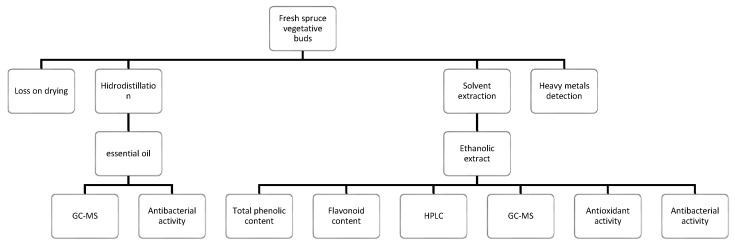
Analysis of the Romanian spruce vegetative buds.

For the other analysis an ethanolic extract was prepared using 1 g of grinded spruce buds (sifted through sieve VI) and 100 mL ethanol 50°, heating the mixture on a water bath at reflux for 30 min, then filtered using a Whatman filter paper. The obtained extract was stored at 4 °C until further analysis was carried out (Figure 9).

### 4.2. Determination of Total Phenolic and Flavonoid Content

The determination of the total phenolic content of the spruce bud extracts was performed using the Folin-Ciocalteau method which allows the estimation of the total phenolic content based on the reaction of the phenolic compounds with the Folin-Ciocalteau reagent, using gallic acid as a standard reference compound for quantification [74,75]. Materials used: Folin–Ciocalteau Reagent (Sigma Chemical, St. Louis, MI, USA), deionized water, 7% sodium carbonate solution, gallic acid (used to create a calibration curve), VWR UV-630 PC Spectrophotometer., Leuven, Belgium. The Folin-Ciocalteau reagent was diluted 10 times.

Folin-Ciocalteau reagent reaction: 1 mL of the spruce bud extract was mixed with 4.5 mL of deionized water and 2,5 mL of diluted Folin-Ciocalteau reagent into separate tubes. After 5 min, 2 mL of 7% sodium carbonate solution was added to the mixture. The samples were then incubated at 25 °C for 30 min. The absorbance of the resulting solutions was measured at 765 nm using the UV-6300 PC spectrophotometer. A calibration curve was prepared using concentrations between 10.00 and 50.00 µg/mL of gallic acid (R^2^ = 0.999). The total phenolic content was determined by extrapolating the absorbance values to the gallic acid standard curve. The results were expressed as milligrams of gallic acid equivalent per gram of dry vegetal product (mg GAE/g DW). The test was done in triplicate.

The total phenolic content (TPC) of extract was determined according to Folin–Ciocalteu’s method using also caffeic acid standard curve (with the equation curve Conc = 77.5789 × Abs; R^2^ = 0.999728). The results were expressed as mg caffeic acid equivalent per g of dry weight (mg CAE/g DW). Measurements were performed in triplicate.

The method used to determine the total flavonoid content, described in the tenth Edition of Romanian Pharmacopoeia, 10th edition [76], is based on the reaction between flavones and aluminum chloride in the presence of sodium acetate, when a yellow-colored complex is formed. This complex has a specific absorbance at a certain wavelength, in this case, at 430 nm, which allows the quantification of the flavone content by measuring the absorbance at this wavelength. A calibration curve using rutin as standard substance was used to determine the concentration of flavonoids in the plant extract (with the equation curve Conc = 25.1359·× Abs; R^2^ = 0.999439). Flavonoid content (TFC) was expressed as milligrams of rutin equivalents/g of dry plant product (mg RE/g DW). The test was done in triplicate.

### 4.3. HPLC Analysis

The analysis of phenolic compounds from the spruce bud extract was also performed by high-performance liquid chromatography (HPLC) using the following equipment and conditions: HPLC Agilent Technologies 1260 Infinity (Santa Clara, US, USA) consisting of a binary pump, thermostatic autosampler, thermostatic compartment for columns, DAD detector, Open Lab CDS Version 2.7 software; Chromatographic column: Kinetex C 18 (150 × 3 mm, 2.6 µm), thermostated at 40 °C. The compounds were eluted with methanol: 0.1% phosphoric acid mobile phase, in a linear gradient from 35% to 65% methanol, in 25 min, with a flow rate of 0.3 mL/min. The signals were acquired at 260 nm. Reference substances were used: quercetin hydrate, kaempferol and kaempferol-3-glucoside (astragalin) (Santa Cruz Biotechnology, Dallas, TX, USA), methanol HPLC, phosphoric acid 85% (Carlo Erba Reagents, Cornaredo, Italy). Stock reference solutions of approx. 1 mg/mL concentration were prepared in methanol, from which dilutions were made in 70% methanol to obtain a mixed solution of approx. 25 µg/mL of each flavonoid compound.

The test solution was obtained by diluting the hydroalcoholic extract of spruce in 70% methanol, in a ratio of 1:5. All analyzed solutions were filtered through 0.22 µm PTFE filters and transferred into autosampler vials. 3 µL of the prepared solutions were injected.

### 4.4. Gas-Chromatographic Analysis

The following equipment and chromatographic conditions were used for GC-MS analysis: GC-MS Trace 1310/TSQ-8000 Evo system (Thermo Scientific, Waltham, MA, USA); CT-Split injector, with split 1:60, injected volume: 1 µL; TG-5SILMS column, 30 m × 0.25 mm × 0.25 µm, flow rate: 1 mL/min; Temperature program/oven: T_0_ = 100 °C (ct = 4 min), with temperature ramps up to 280 °C (ct = 30 min); MS: transfer line: 280 °C, ionization source: 230 °C, range of scanned masses: 40 ÷ 650 u.a.m.; Chromeleon software (v. 7.2.7) and the NIST mass spectra library. The following chemicals and reagents were used: N,O-Bis (trimethylsilyl) trifluoroacetamide (BSTFA) (Supelco, Bellefonte, PA, USA) and HPLC acetonitrile (Carlo Erba Reagents). The liquid sample (50 µL) was evaporated to dryness in the conical derivatization vial, at room temperature. The derivatization of the sample was carried out with 0.5 mL of BSTFA solution and 0.5 mL of acetonitrile, after stapling, at 100 °C for 1 h; the sample was filtered with PTFE 0.2 µm, and the separated derivatized (silylated) compounds were identified with the help of the NIST library.

### 4.5. Heavy Metals Detection

The work utilized a PerkinElmer AAnalyst 800 Atomic Absorption Spectrometer equipped with an AS-800 autosampler and Cooling System, ON, Canada. Only reagents of recognized analytical quality were employed for the analysis, including ultrapure water, 37% HCl (Hydrochloric Acid), 65% HNO₃ (Nitric Acid), H₂O₂ (Hydrogen Peroxide). Concentrated metal standard solutions were prepared from high purity metals, oxides, or non-hygroscopic salts, using water and redistilled nitric or hydrochloric acids. Working standard solutions were created by diluting concentrated metal standard solutions at the time of analysis, with a concentration of 1000 mg/L. A mixture of ammonium hydrogen phosphate and magnesium nitrate was employed as matrix modifiers. For the preparation of the sample, 10 g of fresh plant material were placed in porcelain crucibles. The subsequent steps involved drying and calcination, which were performed using a calcining furnace. The process initiated at 100 °C, and the temperature was gradually adjusted, reaching a maximum of 450 °C. The sample was maintained at this temperature for approximately 20 h. The resulting ash was cooled, treated with 1–3 mL perhydrol, and evaporated on a hot griddle. The crucible was placed back in the oven at a temperature below 200 °C, with the temperature gradually increased to 450 °C, and calcination performed at this temperature for 1–2 h or more. This operation was repeated until the sample was completely calcined. The resulting ash was cooled and treated with 5 mL of 6 mol/L HCl. The solution was allowed to evaporate on a sand bath. The resulting residue was dissolved in 10 mL HNO₃ (0.1 mol/L). After 1.2 h, the solution was injected into the atomic absorption spectrophotometer. A blank sample was simultaneously prepared with mineralization reagents. From the mineralized sample solution, both Pb and Cd could be determined. The working solution for Cd had a concentration of 5 µg/L, and the program automatically diluted it from 1–5 µg/L, resulting in a 5-point calibration curve (1, 2, 3, 4, 5 µg/L). The working solution for Pb had a concentration of 50 µg/L, and the program automatically diluted it from 10–50 µg/L, resulting in a 5-point calibration curve (10, 20, 30, 40, 50 µg/L). Matrix modifications were worked with to decrease the matrix effect of the samples (ammonium acid phosphate and magnesium nitrate mixture), and the calibration curve must have a linearity coefficient 0.995 ≥ R^2^ ≤ 1.

Statistical data included concentration interval (expressed in µg/L), the correlation coefficients of the calibration curve, (R^2^), the limits of detection (LOD), and the limits of quantification.

The concentration of each metal, expressed as mg/kg sample was calculated with the formula:

(1) $$Metal\;concentration\;(mg/kg\;sample)\;=\;\frac{V_{b}\times c}{m_{sample}}$$

where V_b_ is the volume of the volumetric flask where the sample was made (50 mL), C is the concentration of the metal expressed as mg/kg sample, and m_sample_ is the mass of the sampled added to the solution.

### 4.6. Essential Oil Analysis

To know the exactly amount of the essential oil, we have first analyzed the loss on drying and the humidity of the raw vegetal product. For this we have put the aerial parts of the spruce buds in a desiccator, carried out over anhydrous sodium sulfate (R), at atmospheric pressure and room temperature, until a constant mass. Based on the plant mass before and after drying, we have calculated the humidity of the fresh aerial parts of the plant.

The fresh grounded spruce buds were hydro distilled in a closed circuit with a glass Clevenger-type apparatus for 3 h (600 mL distilled water was added to 150 g grinded vegetal product), after a method that provide the volumetric assay of the essential oil, according to the 10th European Pharmacopoeia [77]. The operation was replicated for three times.

The content of essential oil was calculated in % (mL relative to 100 g dried plant) with the formula:

Content of essential oil (yield) = [volume of essential oil (mL)/mass of dry vegetative buds (g)] × 100.(2)

The main components of the essential oil were then identified through a Gas Chromatography-mass Spectrometry (GC-MS) analysis. We have used a gas chromatograph Thermo Electron Corporation, Waltham, MA, USA, Focus with splitter, connected to a mass spectrometer Thermo Electron Corporation DSQII. The column consisted of Macrogol 20000 (Ohio Valey, OH, USA), with film thickness 0.25 μm, of 30 m length and 0.25 mm internal diameter. The mobile phase used was helium with a debit of 1.5 mL min^−1^, while the sample injection volume was 1.0 μL. The temperature in the column increased from 65 °C to 200 °C. The injection volume was of 1μL and the run time 60 min. Quantification was performed by integrating the areas under the curves and the peaks of the chromatograms. Identification of the samples was carried out by comparing the sampled spectral peaks with spectra from a Wiley and NIST database.

### 4.7. Antioxidant Activity

To determine the antioxidant activity, the DPPH method was used. This method for evaluating antioxidant activity is based on the use of the DPPH (2,2-diphenyl-1-picryl-hydrazyl) radical, an organic nitrogen radical with an intense purple color. When a solution of DPPH interacts with an antioxidant capable of donating a proton or electron, a stabilized form of DPPH results and the color changes from purple to pale yellow [78]. For this analysis, 100 mg of DPPH was diluted with methanol in a 200 mL volumetric flask. 1 mL of spruce extract was mixed with 5 mL of diluted DPPH and methanol to 25 mL, then mixed. Using varying volumes of extract (ranging from 0.05 to 0.4 mL), each with a consistent concentration of 253,233 μg/mL of total polyphenols (TPC), as determined by the Folin–Ciocalteu assay, allowes the assessment of the dose–response relationship between the extract and its antioxidant activity. The procedure consisted of mixing different volumes of extract with 3 mL of DPPH solution. After 30 min of incubation in a dark place at room temperature, the absorbance was measured spectrophotometrically at a wavelength of 517 nm. The blank is the reaction mixture that does not contain the test extract. All analyses were conducted in triplicate, indicating the reliability and reproducibility of the results. The radical scavenging capacity of the extract obtained from spruce buds was calculated with the formula:

DPPH scavenging activity % = [(Abs_control_ − Abs_sample_)]/(Abs_control_)] × 100 (3)

where Abs_control_ is the absorbance of diluted DPPH; Abs_sample_ is the absorbance of the sample extracts with DPPH.

To quantify the antioxidant activity, the IC50 value was then determined. This value represents the concentration of the sample required to scavenge 50% of the DPPH radical. The IC50 value was calculated from a plotted graph of radical scavenging activity against the concentration of extracts. A lower IC50 value indicates a stronger antioxidant activity of the extract. Therefore, by analyzing the IC50 values obtained from the dose–response relationship, we can assess the potency of the extract as an antioxidant agent and compare its effectiveness with other compounds or extracts.

### 4.8. Antimicrobial Activity

Antimicrobial activity was tested for the reference strains: Gram-positive (*Staphylococcus aureus* ATCC 25923), Gram-negative (*Escherichia coli* ATCC 25922) and yeast (*Candida albicans* ATCC10231).

To perform biological tests, bacterial strains were grown on a Plate Cound Agar (PCA) culture medium at 37 ± 0.5 °C for 22 ± 2 h. Evaluation of antimicrobial activity was performed by two methods: Kirby-Bauer diffusimetric method and liquid microdilution method.

The Kirby-Bauer diffusimetric metod is considered a simple and rapid method for determining the sensitivity spectrum of the microorganism. There are many factors that can influence the result of an antibiogram, including the strain of microorganism studied (inoculum density, species, and age of the culture), the composition of the culture medium (pH, density and thickness of the medium layer), the technique used and the criteria for interpreting the results. To avoid obtaining erroneous results, the technique was performed under standardized and reproducible conditions according to specifications [79,80].

On the surface of the agarized Muller Hinton medium, seeded “in cloth” with a standardized 0.5 MacFarland inoculum (optical density at λ of 550 nm is 0.125), obtained from the test strain, extracts were applied in 5 µL, 10 µL, 15 µL spots. After 15 min after spot application, plates were thermostatted at 35 ± 2 °C for 16–18 h under aerobic conditions.

Reading and interpretation of the results was done qualitatively only, the presence of any zone of inhibition was interpreted as sensitivity (S) and its absence as resistance (R).

The liquid microdilution method comprises several critical steps for a comprehensive test [81]:

1. Preparation of Microplates: Nutrient Broth liquid culture medium was distributed in 96-well microplates under aseptic conditions, with 100 µL in each well, except for column 1, where 180 µL was distributed.
2. Dispensing of Extracts: 20 µL of the extract was dispensed into each well from A1 to H1.
3. Binary Serial Dilutions: Binary serial dilutions of the tested extracts were performed, up to well 10.
4. Inoculation: Bacterial strains were inoculated up to well 11, with suspensions prepared in sterile physiological water (SFW) at a density of 1.5 × 10^5^ microbial cells/mL. Wells 11 served as a bacterial growth control, while wells 12 acted as a sterility control.
5. Incubation: Samples were incubated at 37 ± 0.5 °C for 22 ± 2 h to facilitate microbial growth.
6. Sampling: After the incubation period, 10 µL was sampled from each well.
7. Inoculation on PCA Medium: The sampled volume from each well was inoculated onto PCA agar plates.
8. Final Incubation: Inoculated plates were further incubated at 37 °C for 18–24 h.

### 4.9. Statistical Data Processing

All experiments were realized in triplicate. Statistical analysis of the data was realized by calculating the Mean Standard Deviation (±SD) and analysis of variance (ANOVA) with XL STAT 2022.4.5 statistics software. The probability value at *p* ≤ 0.05 was considered as statistically significant.

## 5. Conclusions

The present study explored the phytochemical composition and potential biological activities of the alcoholic extract and essential oil of Romanian spruce buds (*Picea abies*).

The investigated species contains in the ethanolic extract significant amounts of polyphenolic compounds, suggesting potential antioxidant properties that could be beneficial for various health applications. The findings of this study affirm that *Picea abies* vegetative buds hold significant potential as a source of shikimic acid. The composition of the Romanian spruce essential oil obtained from vegetative buds has complex chemical profile, which gives an important pharmacological potential that can be used in various pharmaceutical preparations.

Alcoholic extract from Romanian *Picea abies* vegetative buds showed antimicrobial activity of different strengths depending on the strains tested. The most obvious effect was observed against a Gram-positive strain. The essential oil potentiates the antimicrobial effect, for a Gram-positive bacteria and a yeast.

Overall, the results of the study confirm the pharmacological potential of spruce vegetative buds as a source of bioactive compounds with antioxidant, antimicrobial, and potentially other therapeutic properties, such as anti-inflammatory, protective, and antitumoral. These findings support the traditional use of spruce in folk medicine and highlight its potential for further exploration in modern pharmaceutical and healthcare applications.

## Figures and Tables

**Table 1:** Total phenol and flavonoids from *Picea abies* buds.

Extract	TPC * (mg GAE/g DW)	TPC * (mg CAE/g DW)	TFC * (mg RE/g DW)
*Picea abies* buds	25.32 ± 2.65 mg	24.39 ± 1.76 mg	10.54 ± 0.583 mg

* TPC = Total polyphenolic content (mg gallic acid/g DW and mg caffeic acid/g DW); TFC = Total flavonoid content (mg rutin/g DW).

**Table 2:** GC-MS analysis results of plant extracts derivatized with TMS.

Compound	Retention Time RT (min)	Phytochemical Class
Xylitol, 5TMS	14.89	polyol
Shikimic acid, 4TMS	16.77	cyclohexanecarboxylic acid
D-Pinitol, 5TMS	17.11	polyol
Quininic acid, 5TMS	17.76	cyclohexanecarboxylic acid
Galactopyranose, 5TMS	18.48	carbohydrates
D-Glucose, 5TMS	20.06	carbohydrates
Gluconic acid, 6TMS	20.28	polyhydroxycarboxylic acid

**Table 3:** Performance parameters for heavy metal determination.

Metals	Concentration Range (mg/L)	R^2^	LOD (mg/L)	LOQ (mg/L)
**Cadmium**	0.001–0.005	0.9998	0.0003	0.001
**Lead**	0.01–0.05	0.9996	0.003	0.01

**Table 4:** GC-MS analysis of the essential oil.

Peak Name	Molecular Formula	Retention Time (min)	Relative Area (%)
Camphene	C_10_H_16_	5.160	2.36
β-Pinene	C_10_H_16_	6.640	2.68
δ-3-Carene	C_10_H_16_	8.895	4.32
β-Myrcene	C_10_H_16_	10.629	1.63
D-Limonene	C_10_H_16_	12.136	40.43
β-Phelandrene	C_10_H_16_	12.547	0.77
Terpinolene	C_10_H_16_	16.710	0.61
Longifolene	C_15_H_24_	26.890	1.04
Caryophyllene	C_15_H_24_	27.645	3.33
Bornyl acetate	C_12_H_20_O_2_	27.937	0.61
Humulene	C_15_H_24_	30.100	0.81
α-Muurolene	C_15_H_24_	31.849	1.11
δ-Cadinene	C_15_H_24_	32.746	9.95
τ-Cadinol	C_15_H_26_O	43.150	1.31
τ-Muurolol	C_15_H_26_O	43.508	3.37
Cedranol	C_15_H_26_O	43.814	0.75
α-Cadinol	C_15_H_26_O	44.504	12.53
13-Epimanool	C_20_H_34_O	52.384	8.52
ent-Kaurenal	C_20_H_30_O	54.561	1.98
α-Pinene	C_10_H_16_	54.781	0.63
Total identified			98.74

## Data Availability

Data are contained within the article and Supplementary Materials.

## Supplementary Materials

The following supporting information can be downloaded at: https://www.mdpi.com/article/10.3390/molecules29092128/s1, Figure S1: GC-MS chromatogram of the Romanian spruce essential oil.
