# Supplementary figures and images for: Phytochemical Characterization, Antioxidant, and Antimicrobial Activity of the Vegetative Buds from Romanian Spruce, Picea abies (L.) H. Karst
Source: Molecules. 2024 May 3;29(9):2128. doi: 10.3390/molecules29092128 (PMC11085860; doi:10.3390/molecules29092128)

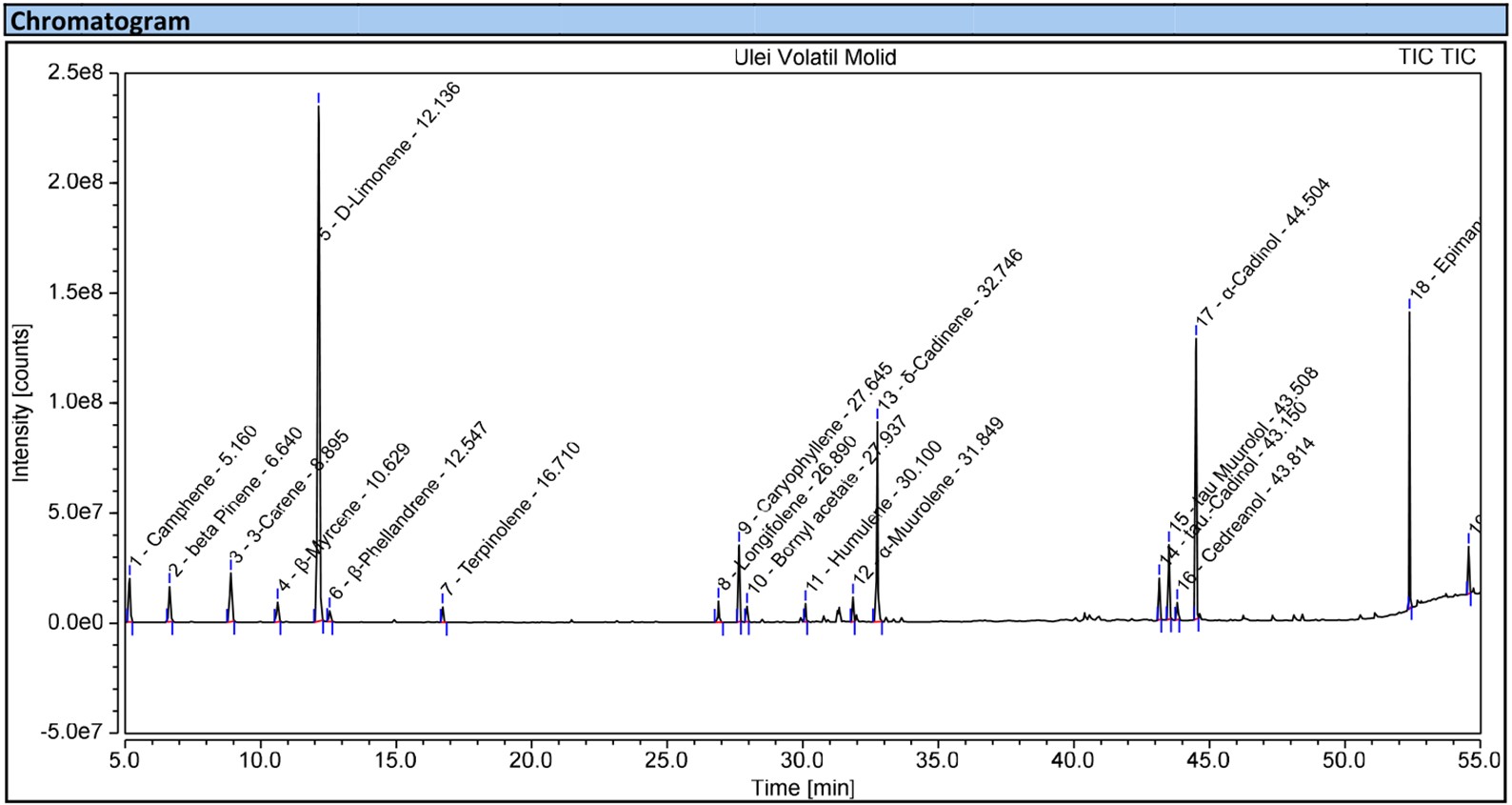

Supplement: Supplementary file 1 [file molecules-29-02128-s001.zip › molecules-2930062-supplementary.jpg]
